# Elucidation of the Intestinal Absorption Mechanism of Loganin in the Human Intestinal Caco-2 Cell Model

**DOI:** 10.1155/2018/8340563

**Published:** 2018-12-23

**Authors:** Renjie Xu, Yichu Yuan, Jia Qi, Jia Zhou, Xiaowen Guo, Jian Zhang, Ruanjuan Zhan

**Affiliations:** ^1^Department of Pharmacy, Xinhua Hospital Affiliated to Shanghai Jiao Tong University School of Medicine, Shanghai200092, China; ^2^Department of Urology, Ren Ji Hospital, School of Medicine, Shanghai Jiao Tong University, Shanghai200127, China; ^3^Department of Pharmacy, The First Affiliated Hospital, Wenzhou Medical University, Wenzhou325035, China

## Abstract

Loganin, iridoid glycosides, is the main bioactive ingredients in the plant* Strychnos nux-vomica L.* and demonstrates various pharmacological effects, though poor oral bioavailability in rats. In this study, the intestinal absorption mechanism of loganin was investigated using the human intestinal Caco-2 cell monolayer model in both the apical-to-basolateral (A-B) and the basolateral-to-apical (B-A) direction; additionally, transport characteristics were systematically investigated at different concentrations, pHs, temperatures, and potential transporters. The absorption permeability (P_app_AB) of loganin, which ranged from 12.17 to 14.78 × 10^−6^cm/s, was high at four tested concentrations (5, 20, 40, and 80*μ*M), while the major permeation mechanism of loganin was found to be passive diffusion with active efflux mediated by multidrug resistance-associated protein (MRP) and breast cancer resistance protein (BCRP). In addition, it was found that loganin was not the substrate of efflux transporter P-glycoprotein (P-gp) since the selective inhibitor (verapamil) of the efflux transporter exhibited little effects on the transport of loganin in the human intestinal Caco-2 cells. Meanwhile, transport from the apical to the basolateral side increased 2.09-fold after addition of a MRP inhibitor and 2.32-fold after addition of a BCRP inhibitor. In summary, our results clearly demonstrate, for the first time, a good permeability of loganin in the human intestinal Caco-2 cell model and elucidate, in detail, the intestinal absorption mechanism and the effects of transporters on iridoid glycosides compounds.

## 1. Introduction

Many traditional Chinese medicines (TCM) have been applied in modern medicine to promote health and prevent disease. TCM have attracted attention due to their distinctive biological activities without toxicity and/or side-effects [1].

**Figure 1:**
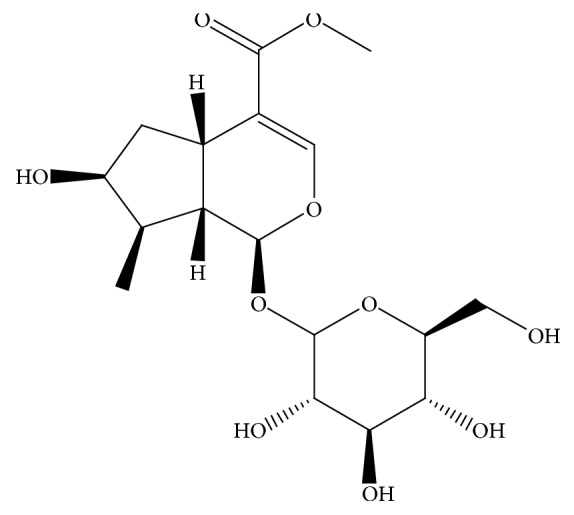
The chemical structure of loganin.

Loganin (Figure 1) is an iridoid glycoside extracted from the plant* Strychnos nux-vomica L.*; additionally, it is also distributed in the plants* Oleander Branch* and* Columelliaceae *[2]. In traditional Chinese medicine,* nux-vomica* is used to enhance limb repair after trauma, whereas loganin is used as a central nervous system (CNS) stimulant in modern medicine. Recent studies have shown that loganin inhibits inflammation [3–5] and protects the kidney [6, 7] and nerves [8–10].

Unfortunately, these pharmacological activities of loganin are curtailed due to its low oral bioavailability [11]. Analogously to other iridoid glycosides, it is well known that loganin exhibits low water solubility which limits its absorption and bioavailability. The intestinal absorption barrier is a major factor controlling the absorption and oral bioavailability of drugs [12], and it is here that the first steps of pharmacokinetics occur after oral intake. Therefore, exploration of the intestinal absorption mechanism of loganin is necessary not only for the* in vivo* pharmacokinetics study but also to provide some key information for their effective delivery systems.

The aim of the present study was to further investigate the intestinal absorption characteristics of loganin by utilizing the Caco-2 human intestinal cells model, an* in vitro* absorption model. Human intestinal Caco-2 cell monolayers have been widely used to determine the permeation rate and to examine the permeation mechanisms of bioactive compounds [13]. These cells also express nutrient and drug transporters that allow for the study of carrier mediated uptake and efflux mechanisms [14]. Different studies demonstrated that* in vivo* absorption could be accurately predicted from the apparent permeation rate (P_app_) across the human intestinal Caco-2 cell monolayers [15–18]. Although the P_app_ values obtained from different laboratories differ, there is a general trend that a high P_app_ implies effective absorption. A published report using the human intestinal Caco-2 cells model for the study of geniposide [19] a structural analog of loganin, showed good intestinal permeability by passive diffusion, chiefly. The present study is devoted for the first time to the investigation of the detailed intestinal absorption mechanism of loganin, and the results will be useful for further studies on this type of compounds* in vitro* and* in vivo*.

## 2. Materials and Methods

### 2.1. Materials

The human colon adenocarcinoma cell line, Caco-2, was purchased from the Cell Bank of the Chinese Academy of Sciences (Shanghai, China). Dulbecco's modified Eagle's medium (DMEM), Hank's buffered salt solution (HBSS), antibiotic solutions (100,000 U/L penicillin and 100,000 mg/L streptomycin), fetal bovine serum (FBS), 100× nonessential amino acids, 100× penicillin and streptomycin, and 0.25% trypsin with ethylenediaminetetraacetic acid (EDTA) were from Invitrogen Corp. (Carlsbad, CA). Transwell permeable polycarbonate inserts (0.4 *μ*m) and 12-well cell culture plates were obtained from Corning. Verapamil, MK 571, indomethacin, benzbromarone, apigenin, sodium vanadate, and cimetidine were obtained from Aladdin Industrial Inc. (Shanghai, China). Loganin (purity > 98.0%) and puerarin (internal standard (IS), purity > 98.0%) were obtained from the National Institute for the Control of Pharmaceutical and Biological Products (Beijing, China). All other reagents were of analytical grade.

### 2.2. Cell Culture

Cells were maintained in DMEM with 10% FBS, 1× nonessential amino acids, and 1× penicillin and streptomycin at 37°C with 5% CO_2_. Cells of passage 35-40 were used in this study to maintain relatively constant cellular phenotypes. The medium was replaced every 2-3 days during incubation. After reaching approximately 80% confluence, the cells were detached using 0.02% ethylenediaminetetraacetic acid (EDTA) and 0.05% trypsin at a density of 5.3×10^4^ cells/cm^2^ on a 60-mm plastic culture dish for the uptake experiment or a 12-mm polyester membrane insert in a 12-well plate for the permeation experiment. The media in the culture plates were changed every two days for the first week after seeding and replaced daily afterward. The integrity of the cell monolayers was examined by measuring the transepithelial electrical resistance (TEER) with a Millicell-ERS-electrode (Millipore Corp, Billerica, MA, USA). After 21-23 days, monolayers with a TEER value (TEER=[TEER_test_- TEER_background_]×area) above 500Ωcm^2^ were used during the transport experiments.

### 2.3. Cytotoxicity Assay

Cell viability was assessed using an MTT assay. Briefly, 0.1 mL of human intestinal Caco-2 cells was seeded onto 96-well plates at a density of 1×10^5^ cells per well in a 96-well plate. The cells were grown in an atmosphere with 5% CO_2_ and 95% relative humidity over approximately 48 h. Subsequently, the culture medium was replaced with HBSS containing different concentrations of loganin (0.1-200 *μ*M); the final concentration of dimethyl sulfoxide (DMSO) in HBSS remained at <1%. The negative control was HBSS containing 1% DMSO. The cells were exposed to the drug for 2 h. Then, 0.2 mL of 0.5mg/mL MTT solution was added to each well and incubated for 4 h in dark. The medium was then removed, the MTT-formazan crystals were solubilized by incubating with 150 *μ*L of DMSO with gentle shaking for 10 min, and absorbance was determined at 490 nm in a Multiskan Spectrum microplate reader (Thermo Labsystems, MA, USA). In each MTT assay, every sample was tested in five replicates and the viability of the nontreated control cells was arbitrarily defined as 100%.

### 2.4. Transport Experiments

Before the experiments, the human intestinal Caco-2 cell monolayers were washed twice with HBSS medium (pH 7.4). The transport experiments were conducted by adding the drug solutions (containing 1% DMSO) to either the apical (AP, 0.5 mL) or basolateral side (BL, 1.0 mL), while the receiving chamber contained the corresponding volume of blank HBSS medium. The monolayers were incubated at 37°C, and 50 *μ*L samples were taken at 15, 30, 45, 60, 90, and 120 min from the acceptor compartment, and the volume was then immediately replaced with50 *μ*L of fresh, prewarmed blank HBSS. TEER measurements for assessing the membrane integrity took place before and after the experiment. The samples were frozen immediately and stored below −80°C before analysis.

The loganin transport (20 *μ*M) at 4°C and 37°C was evaluated in the AP to BL direction to investigate the effect of temperature. The effect of pH on the loganin transport (20 *μ*M) in the AP to BL direction was studied using the following pH combinations for the HBSS in the acceptor/donor compartments: 6.0/7.4 and 7.4/7.4.

Several efflux and influx transporters were investigated for their effects on the transport flux of loganin. One hundred *μ*M verapamil was added to evaluate the selectivity of P-glycoprotein (P-gp) [20]; inhibiting the efflux by multidrug resistance-associated proteins (MRPs) was undertaken by adding 100*μ*M MK571, 50*μ*M benzbromarone, and 200*μ*M indomethacin [21–23]; 25 *μ*M apigenin was used to investigate breast cancer resistance protein (BCRP) [24]; 50 *μ*M sodium vanadate and cimetidine were used as Na^+^/K^+^pump and organic anion transporters (OATs) inhibitors [25].

### 2.5. Sample Processing

In a 1.5mL centrifuge tube, an aliquot of 5 *μ*L of the internal standard working solution (5 ng/mL) was added to 50 *μ*L of collected sample followed by the supplementary addition of 145 *μ*L of acetonitrile (4:1, v/v). After 1 min of vortexing and 10 min centrifugation at 14,000 rpm, the supernatant (2*μ*L) was injected into the UPLC-MS/MS system for direct analysis.

### 2.6. UPLC-MS/MS Analytical Methods

Chromatographic and mass spectrometry detection were performed according to a previous method [11]. Briefly, loganin and puerarin (internal standard, IS) were isolated using a ZORBAX Eclipse Plus C_18_ column (50 mm × 2.1 mm, 1.8 *μ*m) with an API5500 triple-quadrupole mass spectrometer (Applied Biosystems-SCIEX, Concord, Canada). The mobile phase consisted of water-formic acid (100:0.5, v/v) (solvent A) and 100% acetonitrile (solvent B). A gradient program was used for the UPLC separation, with a flow rate of 0.4 ml/min. The initial composition of the mobile phase was 5% solvent B, adjusted to 0-3.1 min (5-63% solvent B), 3.1-3.2 min (63-95% solvent B), 3.2-4.2 (95% solvent B) and 4.2-4.3 min (5% solvent B), and followed by a 1.0 min re-equilibration to the initial condition.

The MS analysis was carried out in multiple reaction monitoring (MRM) mode by monitoring the ion transitions from [M+H]^+^* m/z* 408.2→229.0 for loganin and* m/z* [M+H]^+^ 417.2→296.9 for puerarin (IS). The MS/MS conditions were as follows: ion spray source temperature at 550°C, ionspray voltage (IS) at 5500V; Gas 1 and Gas 2 (nitrogen) at 40 psi, and collision energy (CE) at 12 eV for loganin and 32 eV for IS.

### 2.7. Statistical Analysis

UPLC-MS/MS data acquisition was performed using Analyst 1.5.2 and MultiQuant 2.1.1 software (Applied Biosystems). The data were analyzed by SPSS Software. The apparent permeability coefficient (P_app_) was calculated according to P_app_ = (dQ/dt)/(A·C_0_)

where dQ/dt is the steady-state flux (nmol/s), A is the surface area of the insert (cm^2^); C_0_ is the initial concentration. The efflux ratio (P) was determined by calculating the ratio of P_app_BA versus P_app_AB as in

(1) $$\begin{array}{c} P=\frac{P_{app}BA}{P_{app}AB} \end{array}$$

Statistical analysis was performed with analysis of variance (ANOVA) after confirmation of normal distributions.

## 3. Results and Discussion

### 3.1. Human Intestinal Caco-2 Cell Viability Assay

The viability of human intestinal Caco-2 cells was directly measured by the MTT test to evaluate the cytotoxicity of loganin, and the results were shown in Table 1. As shown in Table 1, the concentrations of loganin ranging from 0.1 to 100 *μ*M were nontoxic to the human intestinal Caco-2 cells after 4 h exposure. When 200 *μ*M of loganin was added, the human intestinal Caco-2 cells were inhibited by 4.67%. Generally, cell survival rates of less than 50% of the controls were considered as the reduction of mitochondrial activity, while an inhibition rate of less than 10% indicated that the compounds at the concentrations were nontoxic to the cells [26]. The results indicated that, in our experimental design, concentrations of approximately 5, 20, 40, and 80*μ*M could be chosen for loganin in the following studies.

### 3.2. Development of LC/MS/MS Method to Quantify Loganin

**Figure 2:**
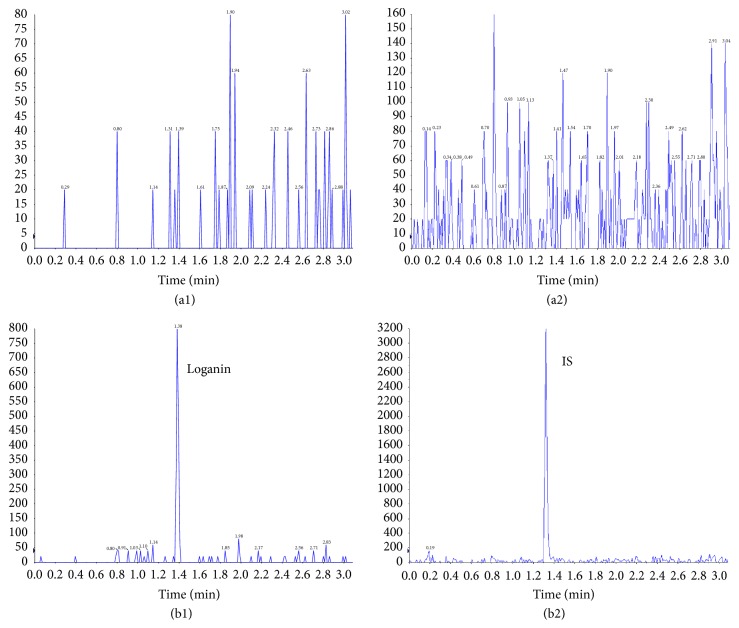
Typical chromatogram of a separation of loganin (1) and IS (2) in fresh blank HBSS (A) and a sample (B) 5 minutes after transport experiments by LC/MS/MS (45.97ng/mL for loganin and 0.08ng/mL for IS).

The retention time for loganin and IS was approximately 1.39 and 1.32 min, respectively. The chromatogram showed baseline separation of loganin and IS without any interference from the endogenous components. The representative chromatograms are presented in Figure 2. A typical calibration curve equation for loganin was y = 0.0496x+0.2201 (R^2^=0.9981), where y represents the ratio of the loganin peak area to the puerarin peak area (x is the concentration of loganin in rat plasma and R is about the correlation coefficient). The lower limit of quantification (LLOQ) in the collected samples was 2 ng/mL and linear ranges from 2 ng/mL to 1750 ng/mL.

### 3.3. Effects of Concentration and Time on Transcellular Transport of Loganin

**Figure 3:**
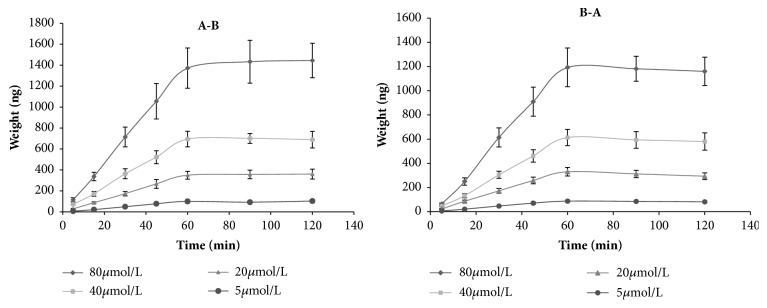
Time Course of loganin transport across human intestinal Caco-2 cell monolayers at different concentrations (n = 3).

The transport of different concentrations (5, 20, 40, and 80*μ*M) of loganin was investigated in both directions, and both the absorptive (P_app_AB) the and secretory (P_app_BA) permeability of loganin were estimated (Figure 3). As shown in Figure 3, P_app_AB of loganin ranged from 12.17 to 14.78×10^−6^cm/s (AP to BL) and from 11.13 to 12.96×10^−6^cm/s (BL to AP). A close correlation between the permeability across human intestinal Caco-2 cell monolayers and the absorption after oral administration* in vivo* has been obtained for several compounds. It is well known that compounds with P_app_ values less than 1×10^−6^cm/s are considered to demonstrate low absorption (<30%), while compounds with P_app_ values between 1×10^−6^ and 1×10^−5^cm/s are considered to have a moderate absorption (30%-70%) and those compounds with P_app_ values of more than 1×10^−5^cm/s are considered to have a high absorption (>70%) [27]. The P_app_ values determined in the present study indicated good intestinal absorption of loganin. At first instance this high permeability observed in the Caco-2 model seems to be inconsistent with the previously reported low oral bioavailability of loganin [11]. Besides its low water solubility, a strong metabolism may be regarded as the major cause of the low oral bioavailability of loganin* in vivo.* In fact, other iridoid glycosides, such as geniposide [28, 29], catalpol [30], and gentiopicroside [31, 32], were effectively metabolized by liver or intestinal microflora. All in all, the* in vivo* processing of drug is complex and additional research is needed to explain the low oral bioavailability.

The transport of loganin in the Caco­2 cell monolayer was plotted versus time at 15, 30, 45, 60, 90, and 120 min for every concentration range. The concentration of loganin increased almost linearly with time within the first 60 min. After 60 min, the concentration gradient between the two sides had greatly decreased, resulting in reduced transport and curves plateau (Figure 3). This indicated that the transport of loganin was driven by a concentration gradient, and passive diffusion represents the main transport mechanism for loganin on both sites.

### 3.4. Effect of pH and Temperature on the Uptake of Loganin by Human Intestinal Caco-2 Cells

**Figure 4:**
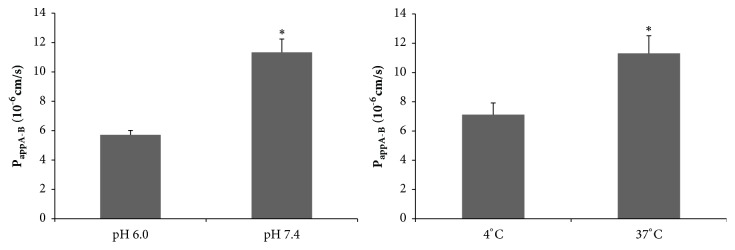
Apparent permeability coefficient (P_app_AB, 60 min) of loganin (20 *μ*M) in the AP-to-BL direction in human intestinal Caco-2 cells previously treated at different PH values and temperatures (n = 3, p < 0.05).

The effects of different pH conditions and temperature on the uptake of loganin by human intestinal Caco-2 cells were investigated, as shown in Figure 4. The P_app_AB values of loganin at pH 7.4 were significantly higher than those at pH 6 (p<0.05), indicating an easier transport of loganin at a higher pH (7.4) than at lower a pH (6.0).

A decrease in temperature reduces cellular metabolism and acts as an inhibitor of energy-dependent transport [33]. As shown in Figure 4, incubation at low temperature (4°C) significantly reduced the uptake of loganin, as P_app_AB decreased from 11.31 × 10^−6^cm/s to 7.12×10^−6^cm/s.

These data show that loganin transport is both pH-dependent and temperature-dependent, indicating that some transporters may be involved in the efflux of loganin. In fact, a pH dependent transport has been reported before for BCRP, being more efficient at lower pH irrespective of the dissociation status of the substrate [34, 35]. It can therefore be assumed that loganin is also a substrate of BCRP. This could be indeed verified in the next experiments.

### 3.5. Effects of Transporters on Transcellular Loganin Transport

Various inhibitors were investigated for their effects on the transport flux of loganin across the human intestinal Caco-2 cells (Table 2). As shown in Table 2, no significant effects were observed in the P_app_ values after pretreatment with 50*μ*M of sodium vanadate or cimetidine, suggesting that the influx transporters like OATs or Na^+^/K^+^ pump contributed little to their transport.

P-glycoprotein (P-gp) has been confirmed as one of the main transporters that influencing drug transport in the intestine [36]. The P-gp inhibitor verapamil may significantly increase the transport of some compounds from the AP to BL side. However, it seemed that loganin was not a substrate of P-gp, as the P_app_AB difference between the verapamil and the control groups was insignificant. In addition to the P-gp transporter, MRP and BCRP transporters are also members of the adenosine triphosphate-binding cassette (ABC) superfamily [37], which is related to the processes of drug absorption and distribution. As shown in Table 2, in the presence of 100*μ*M MK 571, a MRPs inhibitor, the P_app_AB value of 20*μ*M loganin was both almost 2-fold that of the control group, whereas its P_app_BA value was reduced by 66.7%, resulting in a reduction in efflux ratio from 0.9 to 0.14 (p < 0.01). This implied that the MRPs transporters governed the loganin secretion. Earlier observations indicated that Caco-2 cells express more MRP2 and MRP3 than MRP1 and MRP5; moreover, MRP2 and MRP3 were the main apically and basolaterally localized MRPs of the Caco-2 cells, respectively [21, 38]. Therefore, the inhibitory effect of MK-571 implied that MRP2 may be primarily responsible for the loganin efflux in the AP to BL direction. This result was validated by the P_app_AB values when loganin efflux was inhibited by two additional inhibitors, benzbromarone, and indomethacin. Indomethacin is a MRP2 inhibitor that markedly increased the P_app_AB value of loganin (2.0-fold, p < 0.01). Benzbromarone, an inhibitor of MRP2 and MRP3 [21, 23], enhanced loganin transport just like indomethacin (p < 0.01). Seemingly, MRP3 has little effect on the transport of loganin. The result turned out to be that apically localized MRPs especially MRP2 were the main efflux protein in intestinal absorption mechanism of loganin. The observed reduced P_app_BA values in the presence of MK 571, indomethacin, and benzbromarone compared to the control may be attributed to the inhibition of the basolaterally localized MRPs [39, 40].

Moreover, loganin was found to be a substrate of BCRP, as in the presence of apigenin, a BCRP inhibitor, the P_app_AB value was about 2.3-fold that of the control group.

## 4. Conclusions

In the present study, it was shown that the transport of loganin is complex and involves dual processes: (a) passive diffusion as the main absorption mechanism and (b) a major role of transporter mediated active efflux. Efflux transporters BCRP and MRP are vital for loganin transport in the intestine. The elucidated loganin absorption mechanism provides useful information for the study of pharmacokinetics. Further studies are needed to explain the low oral bioavailability about loganin.

## Figures and Tables

**Table 1:** Survival Rates of human intestinal Caco-2 cells treated with loganin. (n = 5).

Concentration (*μ*M)	Survival Rates (%)
	Loganin
**0.1**	107.66±10.9
**1**	108.73±25.4
**5**	106.57±8.3
**10**	106.22±10.9
**50**	103.45±3.3
**100**	101.45±6.5
**200**	95.33±5.3

^a^Data represent the mean ± SD from five replicates.

**Table 2:** Inhibitory effects on bidirectional loganin transport in human intestinal Caco-2 cell monolayers. (n = 3).

Inhibitors	Transporter	concentration (*μ*M)	P_app_AB _(×10^−6^cm/s)_	P_app_BA_(×10^−6^cm/s)_	P	Modulatory effect
**Control**			13.0±0.7	11.7±0.9	0.9	/
***Efflux transporters***						
**Verapamil**	P-gp	100	11.1±1.7	9.3±1.7	0.84	-
**MK 571 **	MRPs	100	27.2±4.1*∗∗*	3.9±0.2*∗*	0.14	+
**Indomethacin **	MRP2	200	24.7±4.8*∗∗*	4.4±1.1*∗*	0.18	+
**Benzbromarone**	MRP2, MRP3	50	29.5±5.9*∗∗*	6.7±0.9*∗*	0.23	+
**Apigenin**	BCRP	25	30.1±6.2*∗∗*	12.3±2.5	0.41	+
***Infflux transporters***						
**Sodium vanadate **	Na^+^/K^+^pump	50	12.3±0.7	9.0±1.1	0.73	-
**Cimetidine**	OATs	50	11.0±1.5	9.9±2.0	0.9	-

*∗*Denotes results significantly different from those of the control experiments (p < 0.05).

*∗∗*Denote results significantly different from those of the control experiments (p < 0.001).

+ Indicates that the inhibitor has significant effect on the loganin transport.

## Data Availability

The data used to support the findings of this study are available from the corresponding author upon request.
